# Anisotropic Graphene Oxide Aerogels for Vegetable Oil Absorption

**DOI:** 10.3390/ma19122680

**Published:** 2026-06-22

**Authors:** Daniel Ordóñez Oviedo, Nelly Maria Rosas-Laverde, Arturo Barjola, Enrique Giménez, Alina Iuliana Pruna

**Affiliations:** 1 Department of Materials, Escuela Politécnica Nacional, Quito 170524, Ecuador; dordonezo13@gmail.com (D.O.O.); nelly.rosas@epn.edu.ec (N.M.R.-L.); 2 Instituto Universitario de Tecnología de Materiales, Universitat Politècnica de València (UPV), Camino de Vera s/n, 46022 Valencia, Spain; arbarrui@doctor.upv.es (A.B.); enrique.gimenez@mcm.upv.es (E.G.)

**Keywords:** aerogels, reduced graphene oxide, EDA, absorption, oil

## Abstract

Oil spills represent a critical environmental challenge. The wastewater treatment with porous sorbents presents the advantage of higher uptake and recyclability. In this work, highly porous and low-density three-dimensional reduced graphene oxide aerogels were obtained by hydrothermal reduction followed by lyophilization. The porosity and reduction degree of the aerogels were controlled by the addition of reducing species, namely ethylenediamine, and hydrothermal conditions. The aerogels were characterized using scanning electron microscopy, Raman spectroscopy, and energy-dispersive X-ray analysis. The sorption measurements were performed with vegetable oils, namely canola and olive oil, at varying operating temperatures. The morphological analysis revealed a well-defined porosity gradient along the aerogel length, along with a functionalization gradient. The sorption performance is highly dependent on their combined action. The maximum gravimetric absorption capacity was about 122 g g^−1^ at room temperature, increasing to 156 g g^−1^ at 60 °C, with the absorption rate increasing from about 1 g g^−1^ s^−1^ to 15 g g^−1^ s^−1^ within 10 s. These results demonstrate that anisotropic gradient aerogels could be obtained by simple tailoring of the synthesis conditions, and such aerogels could benefit the sorption of oils with higher viscosities in terms of rate, pore filling and retention.

## 1. Introduction

At present, one of the most evident problems in water contamination is oil spills resulting from commercial and domestic use [1]. These fluids may originate from different human activities such as household discharges, industrial activities, the automotive sector, and even large spills from oil tankers in the ocean [2]. This type of contamination represents a silent risk to the environment and public health. In the former case, due to the fragility of aquatic ecosystems, which exhibit sensitivity to physical and chemical water parameters that, when altered, promote the migration of marine species to other ecosystems where they cannot always adapt. Regarding public health, it has been shown that degraded oils are precursors of skin cancer and that their ingestion may cause stomach cancer [3]. For these reasons, several studies have focused on the remediation of water contaminated with different types of oils [4].

This type of contamination—water polluted with vegetable oils—is silent but entails serious environmental problems and directly affects aquatic ecosystems [5] due to changes in ecosystem conditions. The affected parameters include pH, salinity, and dissolved oxygen content, among others, causing marine fauna to migrate to other areas and endangering their survival [6]. Furthermore, when vegetable oils reach agricultural soils, they cause degradation and contamination of natural soil nutrients. Nitrogen, phosphorus, potassium, calcium, and magnesium—essential soil nutrients—can be encapsulated upon contact with oils, preventing plants from absorbing them naturally and reducing land productivity [7].

Previous studies referring to organic materials for oil removal, such as recycled and nonwoven wool fibers, reported absorption capacities of about 14 g g^−1^ [8]. Electrochemical treatment has been reported to achieve a removal efficiency greater than 80% of the oil volume in water [9]. Air flotation is another method that has been explored, achieving up to 60% volumetric removal of motor oil [10]. Nevertheless, such methods present varying limitations.

On the other hand, porous materials offer many advantages for oil spill treatment [11]. Amongst these, GO aerogels have attracted considerable attention as sorbent materials for oil spill treatment thanks to their large specific surface area [12], tunable surface chemistry, and exceptional porosity, typically exceeding 90%, which offer improved uptake, selectivity, removal rates, recycling and retention [13]. GO-based materials have been obtained through different methods such as chemical reduction [14], hydrothermal reduction [15], 3D printing [16], and biomimetic pattern-directed assembly methods [17].

The hydrothermal self-assembly of GO into three-dimensional networks represents a scalable and cost-effective fabrication route, where the degree of reduction, the choice of crosslinking or functionalizing agents, and the drying method collectively determine the final pore architecture and surface wettability of the aerogel [18]. Among the functionalizing agents explored, nitrogen-containing compounds such as ethylenediamine (EDA) have proven particularly effective, as they simultaneously promote chemical reduction in GO, mechanically reinforce the matrix [19] and, by nitrogen doping, enhance the oil selectivity in water/oil separation systems [20].

Despite these advances, controlling pore size distribution to optimize both absorption capacity and absorption rate remains a central challenge in the design of GO-based aerogels. Volume shrinkage during self-assembly, layer stacking, and agglomeration of graphene sheets often results in non-uniform pore structures with limited absorption performance for highly viscous liquids [21].

Recent studies have demonstrated that anisotropic and hierarchical pore architectures can significantly reduce flow tortuosity and improve the absorption kinetics of viscous oils compared to aerogels with uniform pore distributions [22]. However, the deliberate introduction of a porosity gradient along the monolith axis—and its systematic correlation with absorption performance—has received limited attention in the literature. Moreover, reports are abundant on the sorption of oils with low viscosity, but average viscosity oil received limited attention.

Freeze-casting offers a promising pathway to achieve such gradients, as the freezing front propagates directionally through the hydrogel, creating a spatial variation in pore size and density that can be exploited to tailor the absorption behavior of different sections of the aerogel monolith [23]. By freeze-casting, excess water is removed. It is known that slow cooling rates result in larger ice crystals and, after drying, larger pore sizes [24], and conversely, fast freezing leads to smaller and homogeneously distributed pores [25]. Increasing porosity generally results in smaller pore sizes and lower capillary forces, which hinder absorption because it becomes more difficult for the fluid to fill all the pore spaces. In contrast, larger pores facilitate fluid transport through the void spaces, and capillary forces assist in filling a greater number of pores within the material [26].

In this study, three-dimensional graphene oxide aerogels with porosity gradients were synthesized by combined hydrothermal synthesis and freeze-casting process [27]. The porosity gradient was enhanced by varying the functionalization degree during the hydrothermal step and by freeze-casting. The performance of the obtained aerogels was tested for the removal of vegetable oils that exhibit average viscosities. To observe the sorption performance of the aerogel with anisotropic aerogels, three sections of the aerogels were tested [28], and the maximum gravimetric absorption capacity and absorption rate were obtained on oils with varying viscosities at ambient temperature and elevated temperature (60 °C) [29]. The enclosed results indicate that anisotropic GO aerogels could be obtained by careful consideration of the synthesis conditions, namely hydrothermal and freeze-casting ones, that could be employed for improved oil spill treatment with respect to more homogeneous ones.

## 2. Materials and Methods

### 2.1. Synthesis of Graphene Oxide Aerogels

For the synthesis of the three-dimensional graphene oxide aerogels, reagent-grade chemicals were used without any prior modification, all of them were purchased from Sigma-Aldrich (Madrid, Spain). The aqueous slurries of graphene oxide nanosheets were supplied by Graphenea Tech Co. (Donostia, Spain, product number 947-768-1). According to the supplier, the mean lateral particle size is 14.6 microns, and the monolayer content (measured in 0.5 mg·mL^−1^ solutions) is over 95%. Ethylenediamine (EDA) was purchased from Sigma-Aldrich (Madrid, Spain) and used as received.

**Figure 1:**
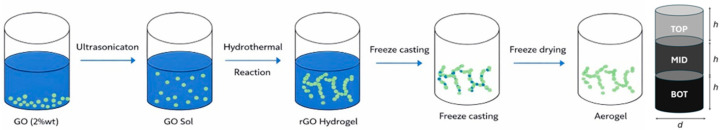
Synthesis methodology.

The graphene oxide (GO) aerogels with porosity gradients were synthesized according to the scheme shown in Figure 1. An aqueous GO dispersion with a concentration of 2 mg mL^−1^ was sonicated for 30 min. Upon the addition of EDA in GO:EDA weight ratio of 1:2.5, the aqueous dispersion was sonicated again for 30 min. The hydrothermal reduction was performed by maintaining the vial with 15 mL of dispersion in a hydrothermal reactor at 140 °C for 12 h. The temperature of 140 °C was selected because it promotes the removal of oxygen-containing functional groups due to the higher thermal energy supplied to the process [30]. A reaction time of 12 h was indicated as sufficient to achieve uniform temperature distribution and remove all or most of the oxygen groups [31]. Additionally, aerogel reduction was performed at a lower temperature of 85 °C for 24 h to assess the effect of functionalization with amine groups by increasing reaction time while supplying lower thermal energy on the sorption performance, considering also their role as spacers in the aerogel matrix [32]. Once the reaction was completed, excess water was removed, and the obtained hydrogel was subjected to directional freeze-casting at −20 °C. After freeze-casting, the hydrogel was subjected to a lyophilization process (Telstar Lyo Quest), which involves applying controlled pressure (0.0015 mbar), time, and temperature conditions (20 °C) to sublimate trapped water ice crystals [12] and obtain the graphene oxide aerogel. As a result, the sublimation of ice crystals formed during freeze-casting leaves behind empty spaces constituting the porous matrix of the aerogel.

To analyze the porosity gradient, three sections of the synthesized aerogel were obtained, as illustrated in Figure 1, the cylindrical aerogel monolith being divided into three equal parts across the length: top—at the interface with air (Top), middle (Mid), and bottom—at the interface with the container (Bot).

### 2.2. Characterization of Graphene Oxide Aerogels

Once the GO aerogels were fabricated, physical characterization was initiated through direct measurements of sample mass and volume [33]. Density, pore volume, porosity, oil occupancy percentage, and gravimetric absorption capacities were calculated using the equations [19,23]: *ρ_a_* = *m_a_*/*v_a_*, *v_p_* = 1/*ρ_a_* − 1/*ρ_g_*, *P* = 1 − *ρ_a_/ρ_g_*, *%O_c_* = 100 × *v_a_*/*v_p_*, *Q_g_* = *m_o_*/*m_a_* where *ρ_a_* is the apparent density of the aerogel; *m_a_* is the aerogel mass; *v_a_* is the aerogel volume; *v_p_* is the pore volume; *ρ_g_* is the density of graphite (2.26 g cm^−3^) [34]; *P* is the porosity of the aerogel, *%O_c_* is the pore occupancy with oil; *Q_g_* is the gravimetric sorption capacity.

To analyze morphology, evolution of pore size, and elemental composition of the GO aerogels, scanning electron microscopy (SEM) was employed using a microscope (JSM-6300, JEOL, Tokyo, Japan) equipped with an energy-dispersive X-ray spectroscopy detector (EDS; Oxford Instruments, Bristol, UK). Raman spectroscopy performed with an Xplora spectrometer equipped with a 532 nm laser (Horiba, Villeneuve-d’Ascq, France) was used to analyze the degree of reduction/defects through the functional groups remaining after GO reduction and amine modification [35].

### 2.3. Vegetable Oil Absorption Performance Measurements

Edible vegetable oils with average viscosities were used, namely canola oil (kinematic viscosity: 78.2 mm^2^ s^−1^) and olive oil (kinematic viscosity: 89 mm^2^ s^−1^). First, the aerogels were allowed to equilibrate in the water for 30 s. Then, the pigmented oil (food-grade dye (Oil Red to facilitate visual observation) was added in increments of pigmented oil (0.1 mL) to distilled water, and its absorption duration was recorded. This procedure was repeated until the aerogel reached its maximum absorption capacity. The absorption capacity was obtained for both given durations (120 s) and as maximum values. Additionally, to establish the effect of temperature on the absorption process, removal tests were performed at 60 °C. The pigmented oil was maintained at the same temperature as the water bath. This approach enabled the determination of improved absorption performance associated with changes in oil viscosity [36]. In this case, the sorption capacity was recorded at 10 s.

## 3. Results and Discussion

**Figure 2:**
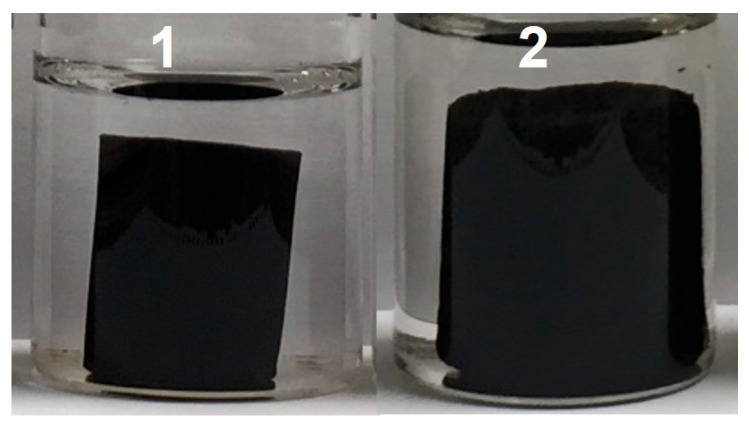
Images of obtained hydrogels: 1—GOA_1_ and 2—GOA_2_.

The reduction in the GO as a function of hydrothermal conditions can be observed from the hydrogel images as shown in Figure 2. Increasing hydrothermal temperature (despite a lower duration of 12 h with respect to 24 h at 85 °C) resulted in approx. 20% volume shrinkage, with contributions from both diameter decrease and height decrease, and it is attributed to the removal of intercalated water molecules and oxygen groups in GO being more efficient at higher synthesis temperature.

**Figure 3:**
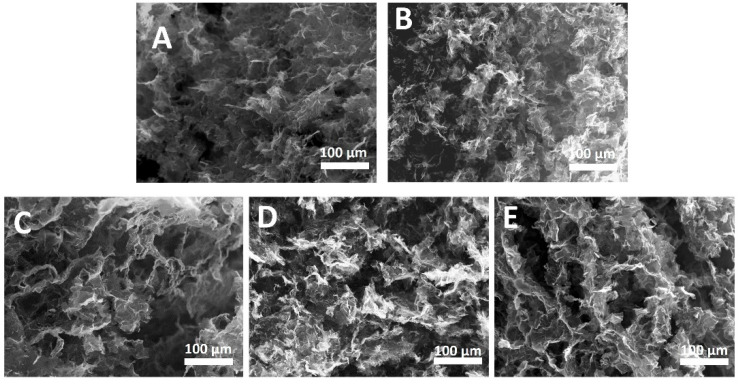
Aerogels (freeze-cast at −80 °C) obtained as function of EDA addition ((**A**,**B**)—without; (**C**–**E**)—with) and hydrothermal synthesis conditions: (**A**,**C**)—synthesized at 85 °C for 24 h; (**B**,**D**)—synthesized at 140 °C for 12 h and (**E**)—synthesized at 165 °C for 12 h.

The hydrogels were subjected to freeze-casting and lyophilization. To observe the evolution of the aerogel microstructure with the freeze-casting and hydrothermal conditions, first the aerogels were obtained in the absence and presence of EDA, followed by lyophilization at −80 °C, that is providing with intermediate cooling rate. The SEM images of the intermediate section of the obtained aerogels are depicted in Figure 3. It was observed that the addition of EDA results in more stacking and smaller sheet size, while the increase in the hydrothermal temperature intensifies these outcomes. The kinetic rate of reduction and functionalization is faster at higher temperatures, resulting in a smaller, highly crosslinked, more homogeneous and dense graphene network. These results agree with the hydrogel volume shrinkage and show the effect of combined reduction by temperature and EDA as a reducing agent.

**Figure 4:**
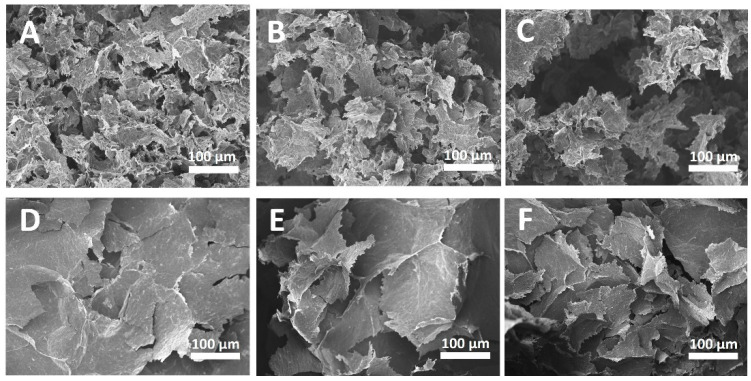
EDA-modified aerogels (freeze-cast at −20 °C) obtained under hydrothermal synthesis conditions: (**A**–**C**)—synthesized at 140 °C for 12 h; (**D**–**F**)—synthesized at 85 °C for 24 h, where (**A**,**D**)—bottom section; (**B**,**E**)—middle section; (**C**,**F**)—top section.

On the other hand, the EDA modified aerogels were obtained in the lower and higher temperature regimes but freeze-cast at a lower rate, which allows for ice crystal growth, which further yields big pores and stacked sheets. The evolution of aerogel characteristics with the combined conditions is depicted in Figure 4. The hydrogels obtained at lower temperatures have a generally lower reduction/functionalization degree, due to a lower rate. In the low-temperature regime, the GO sheets and EDA are allowed to decant to the bottom section, where the reduction will be higher. When coupled with the gradient induced by the freezing, the aerogels behave differently. The one obtained at lower hydrothermal temperature was constituted by sheets that retained partial hydrophilicity and bound water molecules, which act as a structural spacer, showing large pores. Thus, this hydrogel will exhibit stacked sheets at the bottom, pushed by the big ice crystals that were formed using the bound water molecules as seeding sites during the freeze-casting. On the other hand, the hydrogel obtained at higher hydrothermal temperature involves convection together with a faster reduction rate that allows the GO sheets to move upwards, where the pores get bigger as well due to gas evolving during reduction. As the graphene sheets become depleted of oxygen and turn hydrophobic, they stack together. Since the oxygen depletion is faster in this case, a size reduction adds to the stacking, causing the hydrogel to shrink more. Then, upon freeze-casting, the latter aerogel responds more homogeneously across the length, in contrast to the former. As the sheets were packed with fewer water molecules, the ice crystals grew smaller, and the resulting pores were reduced, which is opposite to the 85 °C counterpart, where slower freezing led to larger crystals. On the other hand, closed pores are observed at the top section of the aerogel obtained at the higher temperature. The aerogel density evolution agrees with the hydrogel volume shrinkage. The aerogel density generally increased with the hydrothermal synthesis temperature, from about 8 mg cm^−3^ to about 12 mg cm^−3^. These results consider also the increased mass due to increased functionalization degree with amine groups from EDA.

**Figure 5:**
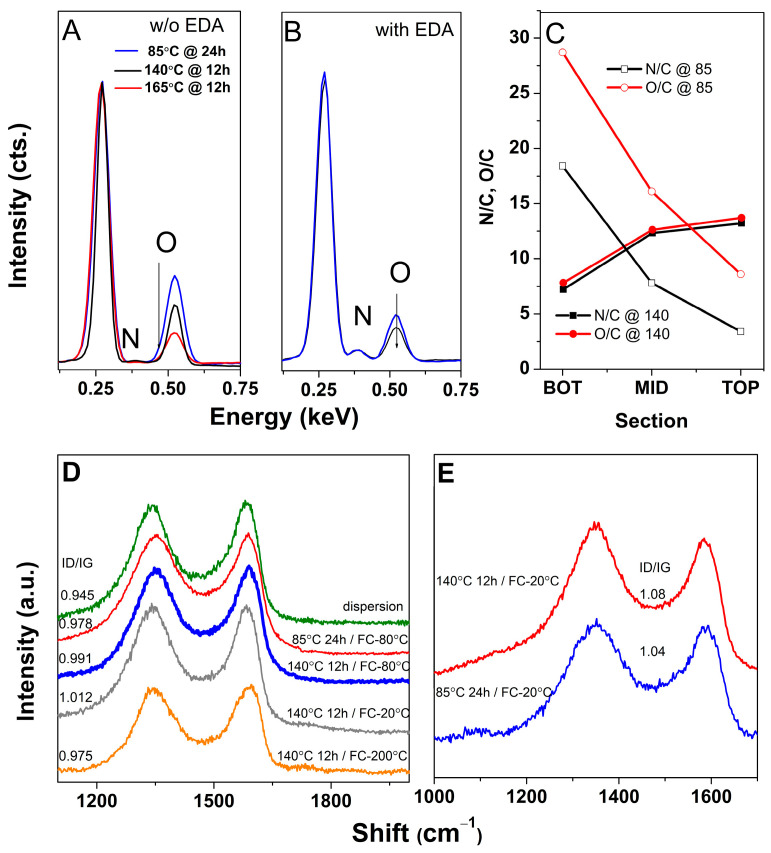
EDAX spectra of the GOA aerogels: (**A**) without EDA; (**B**) with EDA; (**C**) N/C and O/C ratio across the aerogel; Raman spectra of GO dispersion and aerogels obtained without EDA (**D**) and GO aerogels modified with EDA (**E**) as a function of hydrothermal synthesis and freeze-casting temperature [°C]).

In addition to SEM analysis, to evaluate the reduction of oxygen-containing functional groups, EDAX was employed [37]. The EDAX spectra of the middle section of the aerogels are depicted in Figure 5A,B. In the absence of EDA, the removal of oxygen groups is attributed to the hydrothermal reduction, as indicated by a decrease in the oxygen peak as the thermal reduction conditions get harsher, that is, at higher temperatures. Similarly, the aerogels obtained in the presence of EDA have shown a decreasing oxygen contribution with the increase in temperature, attributed to the removal of oxygen groups. The N from the amine groups inserted between the GO sheets increased from 85 °C to 140 °C, indicating an improved functionalization degree. The EDAX analysis, as depicted in Figure 5C, indicates that the functionalization degree and reduction degree markedly decreased from the bottom to the top section for the aerogels obtained at low hydrothermal temperature, which agrees with the previous SEM images indicating a marked gradient in sheet stacking and pore size. On the other hand, the conditions provided by the higher temperature regime (faster kinetics and convection) allow for more homogeneity not only in pore size and sheet size and stacking, as indicated by SEM images, but also in reduction and functionalization degree across the length of the monolith.

Raman spectroscopy was performed on both GO dispersion and the middle section of GO aerogels as a function of the presence of EDA and hydrothermal conditions. The spectra depicted in Figure 5D,E exhibit the typical D and G bands, which represent structural defects and sp^2^ hybridization characteristic of graphene, respectively. The peaks appear at approximately 1349 cm^−1^ for the D band and 1587 cm^−1^ for the G band. The intensity ratios of these bands are presented, enabling the analysis of the reduction in functional groups present in the aerogels [38]. On the one hand, it was observed that the ratio corresponding to the GO nanosheets from the dispersion is the lowest, namely 0.945. Upon reduction by hydrothermal synthesis, the peak ratio increases, especially with increasing synthesis temperature. To observe how the freeze-cast affects the defect degree in the GO aerogels, in the absence of EDA, varying cooling rates were applied for the aerogel reduced in harsher conditions. It was observed that the fast cooling achieved by liquid nitrogen resulted in a lower defect degree, which is associated with smaller ice crystals that do not disrupt the GO sheets heavily in comparison with the other extreme, namely by using the lowest cooling rate in a freezer, which allows the formation of big ice crystals whose sublimation disrupts the sp2 network of the GO. Upon addition of EDA, the functionalization with amine groups leads to a higher I_D_/I_G_ ratio compared to the counterpart aerogels without EDA [39].

**Figure 6:**
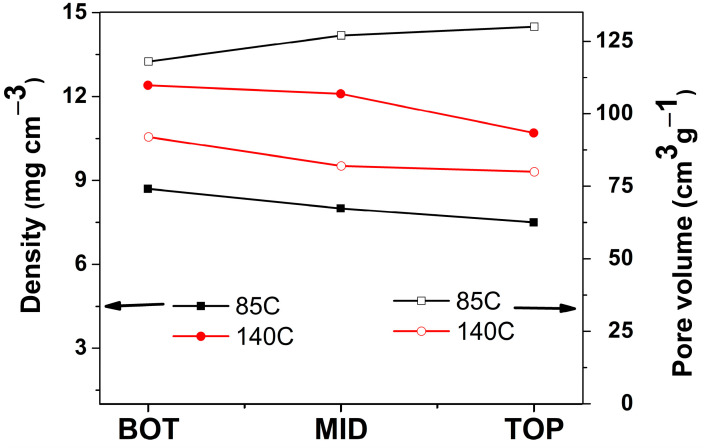
Apparent density of aerogels and pore volume evolution across the aerogel length.

The apparent density of the aerogel sections is further presented in Figure 6. It is shown that it decreased towards the upper section, which can be attributed to the effect of cooling rate, reduction and functionalization and thus, it provides another piece of evidence of a property gradient as a function of the aerogel section [40]. In addition, the pore volume is influenced by spacer content, the freeze-casting method, and the reduction degree. It is shown to decrease with the hydrothermal temperature, which is attributed to the harsher reduction conditions at higher temperatures. The pore volume increased from the bottom region toward the top one for the aerogel obtained at a lower temperature, in agreement with the heavy stacking of sheets at the bottom of the aerogel, as indicated in the SEM analysis. On the other hand, the aerogel obtained at a higher temperature was reduced faster, and its sheet got stacked faster; thus, the hydrothermal reduction and freezing resulted in close-cell walls around pores towards the top section, as indicated by the SEM image. The pore volume for the top section of the latter aerogel is, thus, lower, while the bottom part is slightly increased.

The sorption performance on vegetable oils was first evaluated in terms of pore occupancy with oil, that is, the percentage of the available internal void space that is successfully filled and packed by the oil molecules [41]. Previous studies [42] have demonstrated that porosity gradients in aerogels significantly influence absorption properties. The pores of the aerogel obtained at lower temperature got filled about 65 to 80% from the top to bottom section, which agrees with the large pores towards the bottom that are easier to get occupied. On the other hand, the aerogel obtained at a higher temperature showed a reversed trend for the different oils. The olive oil filled the large pores at the top section of the latter aerogel, reaching a value of 83%, while the bottom one got filled with about 77% of the pore volume. The canola oil, while exhibiting less viscosity than olive oil at room temperature, has a complex molecule that may result in steric hindrance when flooding the aerogel pore; thus, its occupancy at the top section reached only 78% as the aerogel consisted of closed and smaller ones, while towards the bottom it reached 89% as the pores were interconnected.

**Figure 7:**
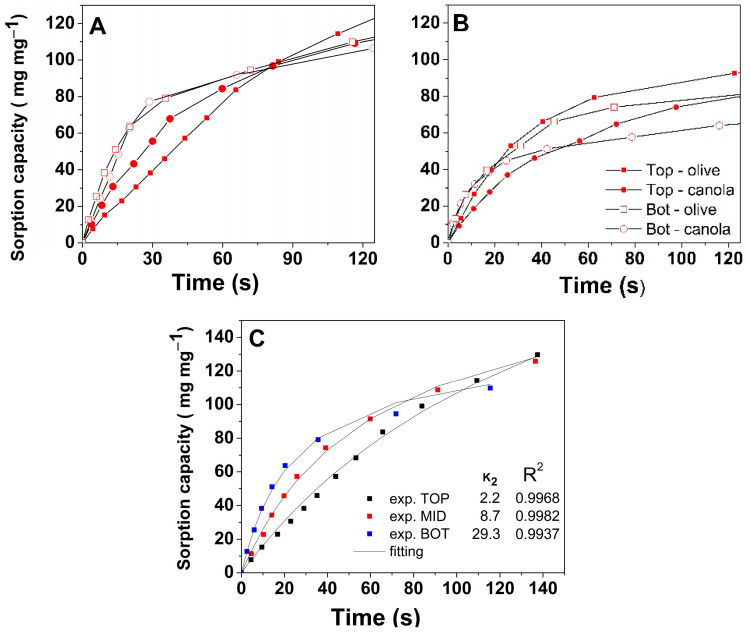
Comparison of absorption capacity kinetics curve (Qg) for aerogels obtained at 85 °C (**A**) and 140 °C (**B**) at ambient temperature. Experimental data and fitting curve with pseudo-second order kinetic model (kinetic rate k_2_ [10^−4^ g g^−1^ s^−1^] (**C**) for the olive oil sorption on the aerogel prepared at low temperature.

The absorption kinetics for vegetable oils on the obtained aerogels are presented in Figure 7. It should be noted that an absorption time of 120 s was considered, as the process occurs rapidly. Figure 7A shows the absorption results for the aerogel obtained at a lower temperature. As observed, the top section of the aerogel results in slower kinetics for the sorption of both oils with respect to the bottom one, in agreement with the SEM that indicated smaller pores. Despite the slow rate of sorption, the top section consists of fewer stacked sheets that offer flexibility; thus, the oils can penetrate the pores and result in higher sorption capacity. On the other hand, the smaller pores at the top section are sensitive to oil viscosity; that is, the oil with higher viscosity (olive) exhibits slower sorption kinetics.

On the other hand, the effects of reduction conditions can be observed in Figure 7B. The aerogel obtained at 140 °C exhibited a top section with large pores, stacked sheets and a bottom one with smaller but interconnected pores. Nevertheless, the pores in the aerogel obtained at higher temperatures are smaller than those obtained at lower temperatures. Regarding the kinetics at the top section, the olive oil exhibited faster uptake, most probably due to its linear molecules, while the canola was uptaken slower, which could be due to the hindrance the molecule may have encountered. Regarding the kinetics at the bottom section, slightly faster uptake was observed, which could be attributed to the interconnection of the pores and improved filling. Regarding the absorption capacity, the highest was exhibited by the top section, attributed to the porosity induced by amine groups and the kinetics of reduction at higher temperature [43]. The amines enable the self-assembly of rGO sheets [12] and result in improved porosity up to a certain limit [30], which represents the basis on which a GO:EDA ratio of 1:2.5 was selected [32]. By comparing the sorption capacity as a function of synthesis temperature, it is observed that the aerogel synthesized at 85 °C exhibited higher values than those obtained at 140 °C. This difference is a consequence of higher and faster reduction that induced sheet stacking; therefore, lower pore volume at higher temperature. In the case of EDA-modified aerogels, the performance may be tailored also considering duration and EDA concentration besides the temperature [44]. The sorption of the oil on the obtained aerogel was then fitted according to first-order kinetic and pseudo-order kinetic models. A higher correlation coefficient indicated the pseudo-order model fits the sorption process better, that is, the rate-limiting step is driven by intermolecular friction, chemical affinity, or heavy steric sharing between the oil and the pore walls. Figure 7C presents the experimental points and the fitting curve according to the pseudo-order kinetic model for the absorption of olive oil onto aerogel obtained at low temperature. As can be seen, good correlations are obtained. The fitting with this model indicates that oil characteristics such as viscosity, density, and molecule sliding beside the pore availability need to be accounted for in the performance.

**Figure 8:**
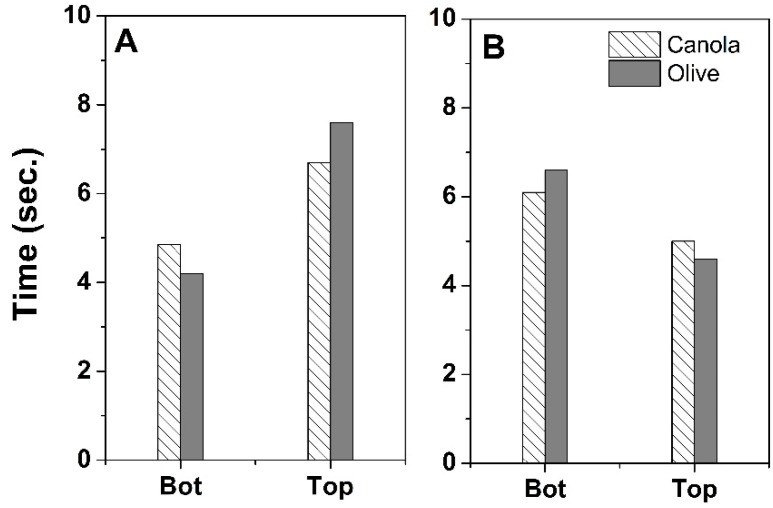
Sorption duration at 60 °C onto aerogels (**A**) prepared at 85 °C and (**B**) 140 °C.

Operating temperature is known to influence absorption performance [45], as increasing operating temperature reduces oil viscosity; thus, making the oils more fluid and improving the pore filling [46]. In this respect, the absorption tests were conducted also at elevated temperature of 60 °C, as shown in Figure 8. In the case of olive oil, the viscosity decreases to 0.0261 ± 0.0003 Pa·s, corresponding to approximately 50% of its value at ambient temperature, while canola oil reaches a viscosity of 0.0188 ± 0.0002 Pa·s [47]. The results indicated improved kinetics and absorption capacity in comparison to the operating ambient temperature. For a faster insight, the duration required to absorb the same amount of oil as that absorbed at room temperature (which was measured at 120 s) was recorded. As can be seen, the larger pores located at the bottom region of the aerogel prepared at lower temperatures exhibit the fastest sorption, especially for the olive oil that has a linear molecule. The opposite region, having smaller pores, requires more time and now the olive’s higher viscosity results in a longer duration than canola [48]. In case of the aerogel prepared at higher temperature, the trend is similar; however, the duration requested for the top region is slightly longer as the pores presented closed walls. On the other hand, the smaller pores at the bottom required less time than the counterpart prepared at a lower temperature, as the homogeneity, interconnection and reduction in the sheets is superior. The results indicated the aerogel synthesized at 85 °C continues to exhibit superior absorption performance compared to the aerogel synthesized at 140 °C for 12 h, with maximum absorption capacities of 156 g g^−1^ and 124 g g^−1^ (TOP region) for canola and olive oil, respectively.

The absorption capacities reported in previous graphene-derived porous sorbent materials vary considerably depending on factors such as pore structure, surface chemistry, density, and especially the physicochemical properties of the absorbed liquid [49,50]. Several studies have demonstrated high sorption capacities for petroleum-derived oils and organic liquids; however, direct comparison between reported values should be performed carefully because absorption performance is strongly influenced by liquid viscosity, density, and testing conditions. Generally, low-viscosity hydrocarbons exhibit faster capillary transport and pore diffusion than more viscous oils, resulting in higher apparent absorption capacities [51].

As shown in Table 1, previously reported graphene-based sorbents exhibited varying absorption capacities for different oils under ambient conditions. In the present work, the EDA-modified GO aerogel exhibited an absorption capacity of 122 g g^−1^ for olive oil at ambient temperature, which is comparable to or higher than that of several previously reported graphene-derived porous materials. This result is particularly relevant considering that olive oil possesses higher viscosity than many petroleum-derived liquids commonly used in sorption studies, which generally hinders capillary penetration and diffusion through porous structures.

Furthermore, the aerogels reached a maximum absorption capacity of 156 g g^−1^ at 60 °C, thanks to improved fluidity. This behavior demonstrates the thermal stability of the aerogel during the absorption process [52,53] and indicates a good performance of the aerogels for uptake of less viscous oils.

**Table 1:** Comparison of oil absorption capacities of graphene-derived porous sorbent materials reported in previous studies and the present work.

Material	Oil	Absorption Capacity (g g^−1^)	Temperature	Ref.
Graphene-based carbon fiber aerogel	Crude Oil	92	Ambient	[49]
Graphene aerogel	Diesel Oil	120.1	Ambient	[30]
Reinforced graphene composite aerogel	Motor Oil	98	Ambient	[50]
Elastic Graphene Aerogel	Vegetable Oil	118	Ambient	[54]
Superhydrophobic graphene aerogel	Vegetable Oil	114	Ambient	[55]
GO:EDA aerogel	Olive Oil	122	Ambient	This work
GO:EDA aerogel	Olive Oil	156	60 °C	This work

Another important aspect of the present study is the incorporation of EDA into the GO aerogel, which, coupled with control of hydrothermal conditions and freeze-casting, results in aerogels with an anisotropic structure. The functionalization process may contribute to improved structural integrity, interlayer interactions, and affinity toward organic compounds, favoring efficient oil uptake behavior.

Anisotropic aerogels have been indicated to improve the uptake with respect to homogeneous aerogels [55], as the rate is faster due to lower flow resistance [56]. The anisotropic structures exhibit other benefits, such as mechanical ones, that yield improved reusability [56,57,58]. In some specific cases, isotropic aerogels may be preferred, such as for heavy viscous oils [59]. Nevertheless, more studies are required to investigate the combined effect of reduction/functionalization and porosity on the sorption rate and capacity of graphene aerogels.

## 4. Conclusions

The aerogel characterization indicated an anisotropic structure across its length. This gradient is obtained in specific synthesis conditions, namely hydrothermal and freeze-casting, and it was found to directly influence the absorption capacity of the aerogels. It was observed that regardless of the synthesis conditions, the aerogels exhibited the highest absorption capacities in the top region (TOP) and the lowest capacities were recorded in the bottom region (BOT) of the aerogels, considering the pores generated by freeze-casting, the reduction/functionalization degree and oil characteristics. The aerogel prepared at a lower temperature exhibited the highest performance for the absorption of vegetable oils. The viscosity and density of the oils were evaluated, confirming that these directly influence the absorption capacity of GO-based aerogels. The sorption of the oils onto the obtained aerogels was found to be fitted by a pseudo-second order kinetic model, indicating the importance of the oil molecule sliding, viscosity and porosity in designing such a sorbent. The results of this paper indicate that anisotropic aerogels can be obtained by simple control of the hydrothermal conditions and freeze-casting process, and they exhibit high potential for oil removal from wastewater.

## Data Availability

The original contributions presented in this study are included in the article. Further inquiries can be directed to the corresponding author.
